# Subclassification of Follicular Neoplasms Recommended by the Japan Thyroid Association Reporting System of Thyroid Cytology

**DOI:** 10.1155/2015/938305

**Published:** 2015-02-04

**Authors:** Kennichi Kakudo, Kaori Kameyama, Mitsuyoshi Hirokawa, Ryohei Katoh, Hirotoshi Nakamura

**Affiliations:** ^1^Department of Pathology, Nara Hospital, Kinki University Faculty of Medicine, Otoda-cho 1248-1, Ikoma, Nara 630-0293, Japan; ^2^Division of Diagnostic Pathology, Keio University School of Medicine, Tokyo 160-8582, Japan; ^3^Department of Pathology, Kuma Hospital, Kobe 650-0011, Japan; ^4^Department of Human Pathology, Yamanashi University School of Medicine, Yamanashi 409-3898, Japan; ^5^Department of Internal Medicine, Kuma Hospital, Kobe 650-0011, Japan

## Abstract

*Background*. The Japan Thyroid Association recently published guidelines for clinical practice for the management of thyroid nodules, which include a diagnostic system for reporting thyroid fine needle aspiration cytology. It is characterized by the subclassification of follicular neoplasms, which is different from other internationally accepted reporting systems. *Materials and Methods*. This study examined observer variability in the subclassification of follicular neoplasms among 4 reviewers using Papanicolaou-stained smear samples from 20 surgically treated patients with indeterminate cytology. *Results*. The favor malignant subcategory had high predictive value of malignancy (risk of malignancy: 60–75%) and good agreement among the 4 reviewers (*κ* = 0.7714). *Conclusion*. These results clearly confirmed that the risk stratification of follicular neoplasms, which was adapted from cytology practice of high-volume thyroid centers in Japan, can provide clinically helpful information to estimate the risk of malignancy and to triage patients for surgery.

## 1. Introduction

The National Cancer Institute (NCI) of the United States of America has proposed a reporting system for thyroid fine needle aspiration (FNA) cytology, so-called Bethesda system, which became an international standard of thyroid cytology [1, 2]. Following this recommendation, the UK Royal College of Pathologists (the UK system) [3, 4] and Italian Societies of Endocrinology and the Italian Society for Anatomic Pathology and Cytology joint with the Italian Division of the International Academy of Pathology (the Italian system) [5, 6] updated their diagnostic schema comparable with the Bethesda system. In 2013, the Japan Thyroid Association (JTA) published guidelines for clinical practice for the management of thyroid nodules, including its diagnostic system for reporting thyroid FNA cytology as shown in Table 1 [7–9], using criteria similar to those used in the Papanicolaou Society recommendation [10], the Bethesda system [1, 2], the UK system [3, 4], and the Italian system [5, 6]. The JTA reporting system of thyroid cytology further recommends risk stratification of follicular neoplasms (FN) into favor benign (low risk: LR), borderline (moderate risk: MR), and favor malignant (high risk: HR), which was adopted from the practice of high-volume thyroid centers in Japan [7]. This study examined observer variation in the subclassification of FN among 4 thyroid experts to validate the usefulness and limitations of this characteristic risk stratification of FN recommended in the JTA guidelines. It is not our purpose to address in detail all the morphological issues of thyroid cytology.

## 2. Materials and Methods

Conventional smear samples of indeterminate diagnosis (*n* = 389) were selected from files (*n* = 3843) from the year 2005 at Ito Hospital, Tokyo, Japan, by one (K. Kameyama) of the authors. Inadequate samples or those with poor preparation and cases under clinical follow-up (no final histologic diagnosis) were excluded. There were 91 (23.4%) surgically treated patients with thyroid nodule under indeterminate cytological diagnoses. These patients visited Ito Hospital in 2005 and underwent diagnostic surgery or curative surgery in the subsequent years between 2005 and 2008. There were 48 cases of benign diagnoses, including 29 follicular adenomas (FAs), 19 adenomatous nodules (AN), and 43 malignant diagnoses, including 13 follicular carcinomas (FTCs), 24 papillary carcinomas (PTCs), 1 poorly differentiated carcinoma, 2 C-cell carcinomas, and 3 malignant lymphomas. The risk of malignancy of these surgically treated patients with indeterminate cytology was 47.3%. Twenty cases of cytological smear samples with follicular patterned lesions were randomly selected by one (K. Kameyama) of the authors and they were further analyzed for subclassification of FN and diagnoses by 4 reviewers, as shown in Table 2.

The present study is a reproducibility study undertaken by 4 reviewers (Kennichi Kakudo, Kaori Kameyama, Mitsuyoshi Hirokawa, and Ryohei Katoh) who have special interest in thyroid pathology, all of whom are members of the Clinical Guideline Committee of the JTA. They were requested to subclassify FN following the new JTA reporting system of thyroid cytology as shown in Table 1. Those 20 cases of smear samples (one representative smear sample each stained by the Papanicolaou method) were circulated among the 4 reviewers without clinical information. The 4 reviewers examined cytological samples independently without exchanging opinions and were unaware of the original cytological diagnoses and final histologic diagnoses.

Because this study was conceived as an audit of cytology performance and the results are anonymized, institutional ethical committee permission was not required for its conduct. Informed consent of all 4 reviewers was obtained for this study protocol.

For statistical analysis of observer concordance, *κ* was first introduced as a measure of the level of agreement between pairs of raters and extended to multiple raters, known as composite *κ* [11, 12]. Composite *κ* statistical analysis was performed by using Data Analysis and Statistical software, version 13 (Stata Press, College Station, Texas, USA). Values of *κ* can be interpreted as follows: 0.00–0.20, slight or very weak agreement; 0.21–0.40, weak to fair agreement; 0.41–0.60, moderate agreement; 0.61–0.80, substantial or good agreement; and values over 0.81, optimal or almost perfect agreement. The chi-square test was used to compare categorical data. Results were considered significant at a *P* value of less than 0.05.

## 3. Results

There were 8 malignant cases (2 FTCs, widely invasive; 4 FTCs, minimally invasive; and 2 PTCs) and 12 benign cases (1 AN and 11 FAs). All diagnoses made by the 4 reviewers on the 20 follicular pattern lesions are shown in Table 2. There were only 6 (30%) cases with a consensus diagnosis among all 4 of the reviewers: 3 in FA and 3 in FTC. Diagnoses of FN and HR were made in only 7 cases (35%), including 4 malignant and 3 benign cases. Composite *κ* statistical analysis clearly demonstrated good concordance for the HR diagnosis in malignant cases (*κ* = 0.7714) (Table 3), while this was not found for the other subcategories in both benign and malignant lesions (*κ* < 0.4). It is of note that there was no interobserver variation in FTCs, widely invasive (*n* = 2), and all 4 reviewers classified the 2 cases into the HR subcategory. It is clear that FTCs, widely invasive, have different degrees of cellular abnormality in our study. In contrast, there were significant disagreements among other types of malignancy and benign lesions, which clearly confirmed that the subclassification of FN is not a definite diagnosis but risk stratification useful for triage patients. Concerning FTC, minimally invasive (*n* = 4), there was only one concordant case among the 4 reviewers and all reviewers made a MR diagnosis in 1 case. There were disagreements between MR and HR in 1 case and between LR and MR in 2 cases. As for PTC (*n* = 2), the 4 reviewers gave 2 diagnoses each, which included 3 LRs, 3 MRs, 1 HR, and 1 suspicious for PTC. However, it is of note that there was no single case in our 20 cases whose subclassification varied among all 3 subcategories. In benign final histologic diagnosis (*n* = 12), there were 9 split diagnoses, including 6 cases between LR and MR and 3 cases between MR and HR, but no case between LR and HR. All 4 reviewers agreed on LR in 1 case and MR in 2 cases. The incidence of LR diagnoses by the 4 reviewers in the 12 benign lesions was 31.3% (15/48) and that of LR diagnoses in the 6 cases of FTC was 16.7% (4/24) (*P* = 0.186). The incidence of HR diagnosis in the 12 benign lesions was 10.4% (5/48) and that of HR in the 6 cases of FTC was 47.1% (10/24) (*P* = 0.002). The incidence of malignancy of the HR was high at 60% for reviewer A, 75% for B, 75% for C, and 75% for D (60–75%). The incidence of malignancy in the MR was lower than that of HR and was 33.3% for reviewer A, 35.7% for reviewer B, 25% for C, and 30.8% for D (25–35.7%). The incidence of malignancy in the LR was lower than that in the HR and was 33.3% for reviewer A, 0% for B, 37.5% for C, and 33.3% for D (0–37.5%). Although the risks of malignancy in the subcategories of FN differed among the 4 reviewers, it is of note that HR cytological diagnosis was significantly correlated with malignant histological diagnosis, and the risk of malignancy (60–75%) was significantly higher than that in the other 2 subcategories (0–35.7%) in our study (*P* = 0.002).

## 4. Discussion

Although the standardization of terminology and diagnostic criteria is important for accurate communication among patients, clinical doctors, and cytopathologists [1–10], there are still differences among reporting systems in thyroid cytology as to how to interpret cytological diagnoses and how to decide on the clinical management of patients. In Japan, cytopathologists attempted to use an internationally accepted reporting system, but it had to be modified to fit our practice. In Japan, all patients with indeterminate cytology undergo further diagnostic procedures, without immediate surgery, to search for higher risk patients who should undergo surgery [7–9, 13–15]. It is because the majority of thyroid carcinomas in indeterminate cytology are indolent and more conservative approaches, other than immediate diagnostic surgery, usually do not create any harm to the patient with malignancy [16–20] and diagnostic surgery to all patients with indeterminate cytology results in risks of unnecessary surgery to the patients with benign nodules, more than 80% of the patients [7–9, 19–22]. The proportion of malignancy found at thyroidectomy from patients with indeterminate cytology in this clinical setting will increase in number, and the malignancy rate of patient with indeterminate cytology in our study was calculated as 47.3% as shown in Materials and Methods. Takezawa et al. from Japan retrospectively analyzed their 1606 cytological samples using Bethesda system, although it was written in Japanese with English abstract, and they identified 115 (7.9%) cases of AUS/FLUS (atypia of undetermined significance/follicular lesions of undetermined significance) and 61 (4.2%) cases of FN/SFN (follicular neoplasms/suspicious for follicular neoplasms). The resection rate of their AUS/FLUS nodules was 30.4% (35/115 cases) and its malignancy rate was 88.6% (31/35 cases), and the resection rate of FN/SFN nodules was 36.0% (22/61 cases) and its malignancy rate was 72.4% (16/22 cases), which were very different from the ranges reported in most of the literatures from Western countries using the Bethesda system or the UK system [1, 2, 20–23]. As it is clear, further triage of patients with indeterminate nodules reduces resection rates and increases malignancy rates in any diagnostic systems including the Bethesda system. This clinical management was also true in some other countries [24–27], as Crippa and Dina commented in a letter to an editor that thyroid cytology is the most important but not the only important deciding factor and therefore it must be integrated with other diagnostic procedures [24]. We propose that future thyroid cytology classification schemes should reconsider clinical managements of indeterminate categories and how to reduce unnecessary surgeries for patients with benign thyroid nodules [7–9].

The risk stratification of follicular pattern lesions into 3 subcategories (cellular follicular lesion, FN favor benign, and FN favor malignant) was suggested by the Papanicolaou Society in 1996 [10], but it did not become popular in thyroid cytology worldwide, apart from Japan [8, 9]. There have been some reports in the literature on risk stratification of FN [10, 28–34]. Kelman et al. reported that 31/52 (60%) nodules with nuclear atypia consistent with FN were malignant [28] and it was 4/9 (44.4%) in FN with atypia by Goldstein et al. [29]. Pagni et al. reported that atypical proliferation was more often malignant than follicular group (53% versus 19%) in their indeterminate category (Tir3/Thy3) [30]. Some researchers pointed out that the malignancy rate of FN without atypia is low and assessment later on could be an alternative approach [31, 32] and patients with high-risk cytological features such as nuclear overlapping (crowding) should be advised to have a surgical intervention [31–33].

Gerhard and da Cunha Santos using the Papanicolaou Society guidelines studied reproducibility between 2 observers in 97 diagnoses [35]. They reported a substantial level of diagnostic interobserver (*κ* = 0.71) and intraobserver (*κ* = 0.66) reproducibility, although interobserver disagreement in the cytological diagnosis occurred in 23 cases (24.7%) and 18 (41.7%) of them were for FN [35]. In an interobserver reproducibility study using the UK system, Kocjan et al. reported that the *κ* statistic was very poor (0.11) for the Thy3a category and that for Thy4 was 0.17, in contrast to moderate to good agreement for Thy1 (0.69), Thy2 (0.55), Thy3f (0.51), and Thy5 (0.61) [36].

The observer variation of FN in our study occurred more often between LR and MR (9 cases, 45%), followed by between MR and HR (5 cases, 25%), but it is remarkable to note that none occurred between LR and HR. In other words, discordance is limited to between LR and MR or MR and HR, so we may conclude that the MR subcategory has an essential role in minimizing discordance between LR and HR. Another choice of subclassification of follicular neoplasms would be two categories (low cancer risk and high cancer risk) [30] instead of three categories (LR, MR, and HR), and this modification (two categories) is also described as acceptable in the reporting system recommended by the Japan Thyroid Association [7, 8]. The second conclusion we may draw is that HR in the JTA system is a powerful cytological subcategory to be used for the triage of patients for diagnostic surgery because the risk of malignancy of HR is high (60–75%) equivalent to suspicious for malignancy category in the Bethesda system, with good concordance among the 4 reviewers (*κ* = 0.7714).

Abele and Levine reported their rate of indeterminate category to be 5% of 51,000 adequate FNAs and they suggested that the national rate of 15% was in large part due to overdiagnosis [37]. This significant difference in ratio of indeterminate categories may be due to experience of thyroid cytology and not patients' background. Clary et al. commented in their interobserver variability study that some pathologists make greater use of indeterminate categories such as follicular lesion, favor nonneoplastic or follicular lesion, and favor neoplastic lesion, whereas others show more definitive categorization into benign and neoplastic groups [38]. Cibas et al. also stated in their report on interobserver variability that cytopathologists with experience of thyroid cytopathology are more likely to make a definitive interpretation (i.e., benign or malignant) [21]. This tendency was seen in our present study in which reviewers B and D (whose indeterminate diagnosis rates are about 15% in their practice) made MR diagnosis more often (70% and 65%, resp.) and LR diagnosis less frequently (10% and 15%, resp.) than those of reviewers A and C (whose indeterminate diagnosis rates are about 5% in their practice). The difference in the prevalence of benign and FN/SFN may explain the different rates of LR (low risk) and MR (moderate risk) among reviews in our study, because incidence of one category may expand or contract depending on the rates of other categories [22]. Some FN/SFN lesions with benign pattern would be classified as benign by different authors and the incidence of FN/SFN of Bethesda system in recent 7 series varied from 1.5 to 9.7% and that of benign category was between 54 and 77.4% summarized by Ohori and Schoedel [22].

Rapid development of molecular analyses on thyroid cytology may lead us possibly in the near future to more accurately identify patients who should be referred to surgery. Until that time comes, thyroid FNA cytology remains a main stay in the management of patients with thyroid nodules integrated with other clinical tests, such as ultrasound image diagnosis.

As a conclusion, thyroid cytology recommended by the JTA is characterized by subclassification of FN. The HR subcategory has a high predictive value of malignancy and good agreement among the 4 reviewers which is clinically helpful to triage patients for surgery. We believe that patients with cytological diagnosis of HR subcategory of FN in the JTA system should be surgically treated especially if other risk factors coexisted. On the other hand, patients with LR or MR category should not be immediately sent to operation room unless other risk factors exist. Therefore, surgical resection rate of indeterminate category is low in Japan usually less than 50%, particularly in cases with FN.

## Figures and Tables

**Table 1:** Cytological reporting system recommended by the Japan Thyroid Association guidelines for clinical practice on the management of thyroid nodules, 2013.

Diagnostic category	Risk of malignancy
① Inadequate (nondiagnostic)	10%
② Normal or benign	<1%
③ Indeterminate	
(A) Follicular neoplasms (follicular pattern lesions)	
(A-1) Favor benign	5–15%
(A-2) Borderline	15–30%
(A-3) Favor malignant	40–60%
(B) Others	40–60%
④ Malignancy suspected (not conclusive for malignancy)	>80%
⑤ Malignancy	>99%

**Table 2:** Observer variability on 20 follicular pattern lesions by 4 reviewers.

Histological diagnoses	Reviewer			
	A	B	C	D
AN	1	2	1	2
FA	1	1	1	1
FA	1	2	1	2
FA	1	2	1	2
FA	1	2	2	1
FA	2	1	1	2
FA	1	2	2	2
FA	2	2	2	2
FA	2	2	2	2
FA	3	2	2	2
FA	3	2	3	2
FA	2	3	2	3
FTC, minimally	1	2	1	2
FTC, minimally	1	2	1	2
FTC, minimally	2	2	2	2
FTC, minimally	3	2	3	2
FTC, widely	3	3	3	3
FTC, widely	3	3	3	3
PTC	1	2	1	1
PTC	2	3	2	3^*^

Score 1: follicular neoplasm, favor benign; Score 2: follicular neoplasm, borderline; Score 3: follicular neoplasm, favor malignant; and Score 3^*^: favor malignant but papillary carcinoma was suspected.

AN: adenomatous nodule; FA: follicular adenoma; FTC, minimally: follicular carcinoma, minimally invasive; FTC, widely: follicular carcinoma, widely invasive; and PTC: papillary carcinoma.

**Table 3:** Observer variability among 4 reviewers in malignant thyroid diseases.

Score	*κ*	*Z*	Prob > *Z*
1	0.2000	1.20	0.1151
2	0.3143	1.89	0.0297
3	0.7714	4.63	0.0000

Score 1: follicular neoplasm, favor benign; Score 2: follicular neoplasm, borderline; Score 3: follicular neoplasm, favor malignant.
